# Prophylactic three-dimensional funnel mesh for prevention of parastomal hernia: propensity-score-matched cohort study

**DOI:** 10.1093/bjsopen/zrag072

**Published:** 2026-06-08

**Authors:** Jacques-Emmanuel Saadoun, Eleftherios Gialamas, Alexandre Balaphas, Cécile de Chaisemartin, Hélène Meillat, Bernard Lelong, Christian Toso, Emilie Liot, Jérémy Meyer, Guillaume Meurette, Frédéric Ris

**Affiliations:** Division of Digestive Surgery, University Hospitals of Geneva, Geneva, Switzerland; Department of Digestive and Surgical Oncology, Institut Paoli-Calmettes, Marseille, France; Division of Digestive Surgery, University Hospitals of Geneva, Geneva, Switzerland; Medical School, University of Geneva, Geneva, Switzerland; Division of Digestive Surgery, University Hospitals of Geneva, Geneva, Switzerland; Medical School, University of Geneva, Geneva, Switzerland; Department of Digestive and Surgical Oncology, Institut Paoli-Calmettes, Marseille, France; Department of Digestive and Surgical Oncology, Institut Paoli-Calmettes, Marseille, France; Department of Digestive and Surgical Oncology, Institut Paoli-Calmettes, Marseille, France; Division of Digestive Surgery, University Hospitals of Geneva, Geneva, Switzerland; Medical School, University of Geneva, Geneva, Switzerland; Division of Digestive Surgery, University Hospitals of Geneva, Geneva, Switzerland; Medical School, University of Geneva, Geneva, Switzerland; Division of Digestive Surgery, University Hospitals of Geneva, Geneva, Switzerland; Medical School, University of Geneva, Geneva, Switzerland; Division of Digestive Surgery, University Hospitals of Geneva, Geneva, Switzerland; Medical School, University of Geneva, Geneva, Switzerland; Division of Digestive Surgery, University Hospitals of Geneva, Geneva, Switzerland; Medical School, University of Geneva, Geneva, Switzerland

**Keywords:** prophylactic mesh, permanent stoma, propensity score matching

Parastomal hernia (PSH) is a frequent long-term complication following permanent end colostomy (PEC), with reported incidences up to 80%^1^. Beyond its incidence, PSH significantly impacts quality of life and remains difficult to treat, with high recurrence rates after repair. Preventive strategies are therefore essential, yet the optimal approach remains debated. Considering this problem, funnel-shaped three-dimensional (3D) meshes have emerged as a promising alternative, since Berger first described the technique^2^. This design stabilizes the bowel loop and reduces local tension.

A retrospective study was undertaken comparing PSH incidence and postoperative complications after PEC with or without prophylactic funnel-shaped 3D mesh reinforcement (DynaMesh^®^-IPST; FEG-Textiltechnik, Aachen, Germany). Consecutive adult patients undergoing elective PEC in a tertiary referral centre between 2013 and 2024 were included. Procedures comprised extralevator abdominoperineal excision, low Hartmann procedure, Hartmann procedure, and pelvic exenteration for benign or malignant disease. Patients were divided according to prophylactic intraperitoneal 3D funnel mesh placement. Mesh use was not systematic and depended on progressive adoption, surgeon preference, and intraoperative considerations. Propensity score matching (1 : 1) was performed to reduce selection bias using clinically relevant variables, including age, sex, body mass index, American Society of Anesthesiologists score, neoadjuvant treatment, surgical approach, and type of procedure.

After matching, 112 patients were analysed (56 per group; *Fig. S1*), with balanced baseline characteristics. PSH occurred in six patients in the IPST group, compared with 34 patients in the non-IPST group (11% *versus* 61%, respectively; *P* < 0.001; *Table 1*). Kaplan–Meier analysis demonstrated an early and sustained separation between groups, suggesting that a substantial proportion of PSH developed during the first year after surgery (*Figs S2* and *S3*).

**Table 1:** Baseline characteristics, surgical variables, and postoperative outcomes before and after PSM in patients undergoing permanent end colostomy with or without prophylactic IPST mesh

	Before PSM			After PSM		
	Non-IPST (*n* = 97)	IPST (*n* = 56)	P*	Non-IPST (*n* = 56)	IPST (*n* = 56)	P*
**Demographics**						
Sex						
Male	64 (66%)	27 (48%)	0.031	34 (61%)	27 (48%)	0.200
Female	33 (34%)	29 (52%)		22 (39%)	29 (52%)	
Age (years), median (i.q.r.)	69 (59–77)	70 (58–75)	0.800†	70 (62–78)	70 (58–75)	0.500†
ASA grade			0.200			0.300
I–II	37 (38%)	27 (48%)		22 (39%)	27 (48%)	
III–IV	60 (62%)	29 (52%)		34 (61%)	29 (52%)	
BMI (kg/m^2^), median (i.q.r.)	25.3 (22–28)	24.8 (22–27)	0.400†	24.7 (22–28)	24.8 (22–27)	0.900†
Smoker	42 (43%)	27 (48%)	0.600	24 (43%)	27 (48%)	0.600
Active smoker	25 (26%)	20 (36%)	0.200	14 (25%)	20 (36%)	0.200
Co-morbidity						
Hypertension	38 (39%)	29 (52%)	0.130	24 (43%)	29 (52%)	0.300
Cardiovascular	13 (13%)	10 (18%)	0.500	11 (20%)	10 (18%)	0.800
Diabetes	20 (21%)	10 (18%)	0.700	12 (21%)	10 (18%)	0.600
Chronic renal disease	13 (13%)	7 (13%)	0.900	9 (16%)	7 (13%)	0.600
Cirrhosis	9 (9%)	3 (5%)	0.500‡	5 (9%)	3 (5%)	0.700‡
COPD	6 (6%)	5 (9%)	0.500‡	5 (9%)	5 (9%)	> 0.900‡
Previous abdominal surgery	41 (42%)	26 (46%)	0.600	24 (43%)	26 (46%)	0.700
Surgical indication			0.140‡			0.700‡
Adenocarcinoma	67 (69%)	44 (80%)		45 (80%)	42 (75%)	
Squamous cell carcinoma	6 (6%)	6 (11%)		4 (7%)	5 (9%)	
Diverticular disease	7 (7%)	2 (4%)		3 (6%)	2 (4%)	
Genitourinary cancer	6 (6%)	0 (0%)		1 (2%)	4 (7%)	
Others	11 (11%)	3 (5%)		3 (6%)	3 (5%)	
Neoadjuvant therapy	53 (55%)	40 (71%)	0.040	37 (66%)	40 (71%)	0.500
Irradiation	48 (49%)	39 (70%)	0.015	34 (61%)	39 (70%)	0.300
**Surgery characteristics**						
Surgical procedure			< 0.001			0.500
ELAPE	36 (37%)	39 (70%)		32 (57%)	39 (70%)	
Low Hartmann	31 (32%)	8 (14%)		12 (21%)	8 (14%)	
Hartmann	16 (17%)	2 (3%)		4 (7%)	2 (3%)	
Pelvic exenteration	14 (14%)	7 (13%)		8 (14%)	7 (13%)	
Surgical approach			< 0.001			0.900
Open	43 (44%)	9 (16%)		12 (21%)	9 (16%)	
Laparoscopic	51 (53%)	44 (79%)		41 (73%)	44 (79%)	
Robot	3 (3%)	3 (5%)		3 (5%)	3 (5%)	
**Postoperative outcomes within 90 days**						
Clavien–Dindo complications			0.200			0.300
Grade I	6 (6%)	2 (4%)		3 (5%)	2 (4%)	
Grade II	35 (36%)	19 (34%)		26 (46%)	20 (36%)	
Grade III	12 (13%)	15 (27%)		5 (9%)	13 (23%)	
Grade IV	2 (2%)	1 (2%)		1 (2%)	1 (2%)	
Reintervention	8 (8%)	16 (29%)	< 0.001	4 (7%)	16 (29%)	0.003
**Main outcome and follow-up**						
PSH	56 (58%)	6 (11%)	< 0.001	34 (61%)	6 (11%)	< 0.001
Time to PSH occurrence (days), median (i.q.r.)	373 (220–737)	886 (475–1069)	0.120†	466 (253–778)	886 (475–1069)	0.200†

Values are *n* (%) unless otherwise stated. PSM, propensity score matching; IPST, DynaMesh^®^-IPST (FEG-Textiltechnik, Aachen, Germany). i.q.r., interquartile range; ASA, American Society of Anesthesiologists; BMI, body mass index; COPD, chronic obstructive pulmonary disease; ELAPE, extralevator abdominoperineal excision; PSH, parastomal hernia. *Pearson’s χ^2^ test, except †Wilcoxon rank sum test and ‡Fisher’s exact test.

Overall postoperative morbidity did not differ between groups, and no mesh-related complications were observed. However, reintervention rates were higher in the IPST than non-IPST group (29% *versus* 7%; *P* = 0.003), mainly driven by perineal wound dehiscence. This difference may reflect residual confounding despite matching, particularly the higher proportion of extralevator procedures and pelvic irradiation, both of which are recognized risk factors for perineal morbidity.

Previous randomized trials evaluating prophylactic mesh in PEC have reported conflicting results, particularly with retrorectus techniques^3,4^. More recently, a randomized trial evaluating funnel-shaped intraperitoneal mesh demonstrated a significant reduction in PSH without increased morbidity^5^. The findings of the present study are consistent with these data and support the potential benefit of this approach in routine practice. However, the non-systematic use of mesh in the present cohort reflects real-world decision-making and introduces an inherent selection bias. In this context, the results reinforce the need to better define which patients may derive the greatest benefit from prophylactic mesh placement.

Long-term safety of intraperitoneal mesh remains an important consideration, particularly in oncological patients. In the present cohort, no mesh-related complications were identified during follow-up.

This study has several limitations. The retrospective design and non-systematic use of mesh introduce an inherent selection bias, limiting causal inference, although propensity score matching was used to mitigate this effect. In addition, this was a single-centre study, which may limit generalizability.

Prophylactic IPST mesh placement was associated with a marked reduction in PSH rates after PEC, without increased overall postoperative morbidity. These findings support a potential role while highlighting the need for careful patient selection. Further prospective studies are needed to confirm these results and better define optimal indications.

## Supplementary Material

zrag072_Supplementary_Data

## Data Availability

The data underlying this article will be shared upon reasonable request to the corresponding author.
