# Supplementary material for: Prophylactic three-dimensional funnel mesh for prevention of parastomal hernia: propensity-score-matched cohort study
Source: BJS Open. 2026 Jun 8;10(3):zrag072. doi: 10.1093/bjsopen/zrag072 (PMC13245723; doi:10.1093/bjsopen/zrag072)
Supplement: zrag072_Supplementary_Data [file zrag072_supplementary_data.docx]

**Prophylactic 3D funnel mesh for prevention of parastomal hernia: propensity-matched cohort study**

Jacques-Emmanuel Saadoun^1,3^ MD MSc, Eleftherios Gialamas^1,2^ MD MSc, Alexandre Balaphas^1,2^ MD PhD, Cécile de Chaisemartin^3^ MD, Hélène Meillat^3^ MD, Bernard Lelong^3^ MD, Christian Toso^1,2^ MD PhD, Emilie Liot^1,2^ MD, Jérémy Meyer^1,2^ MD PhD, Guillaume Meurette^1,2^ MD PhD, and Frédéric Ris^1,2^ MD PhD

^1^Division of Digestive Surgery, University Hospitals of Geneva, Genève, Switzerland

^2^Medical School, University of Geneva, Geneva, Switzerland.

^3^Department of Digestive and Surgical Oncology, Institut Paoli-Calmettes, Marseille, France.

**Corresponding author.** Jacques-Emmanuel SAADOUN, 232 boulevard sainte marguerite, 13009 Marseille, France ; [Jacques.saadoun@gmail.com](mailto:Jacques.saadoun@gmail.com) ; **ORCID ID : 0000-0001-6032-9172**;

**Supplementary Materials - Index**

| **Supplementary Methods** |  |
| --- | --- |
| STROBE Check list | *page 2* |
| **Supplementary Figures and Tables** |  |
| Flow chart | *page 3* |
| Kaplan Meier curves | *page 4* |
|  |  |

**Supplementary Methods**

|  | Item No | Recommendation |
| --- | --- | --- |
| **Title and abstract** | 1 | (*a*) Indicate the study’s design with a commonly used term in the title or the abstract |
|  |  | (*b*) Provide in the abstract an informative and balanced summary of what was done and what was found |
| Introduction | | |
| Background/rationale | 2 | Explain the scientific background and rationale for the investigation being reported |
| Objectives | 3 | State specific objectives, including any prespecified hypotheses |
| Methods | | |
| Study design | 4 | Present key elements of study design early in the paper |
| Setting | 5 | Describe the setting, locations, and relevant dates, including periods of recruitment, exposure, follow-up, and data collection |
| Participants | 6 | (*a*) Give the eligibility criteria, and the sources and methods of case ascertainment and control selection. Give the rationale for the choice of cases and controls |
|  |  | (*b*) For matched studies, give matching criteria and the number of controls per case |
| Variables | 7 | Clearly define all outcomes, exposures, predictors, potential confounders, and effect modifiers. Give diagnostic criteria, if applicable |
| Data sources/ measurement | 8* | For each variable of interest, give sources of data and details of methods of assessment (measurement). Describe comparability of assessment methods if there is more than one group |
| Bias | 9 | Describe any efforts to address potential sources of bias |
| Study size | 10 | Explain how the study size was arrived at |
| Quantitative variables | 11 | Explain how quantitative variables were handled in the analyses. If applicable, describe which groupings were chosen and why |
| Statistical methods | 12 | (*a*) Describe all statistical methods, including those used to control for confounding |
|  |  | (*b*) Describe any methods used to examine subgroups and interactions |
|  |  | (*c*) Explain how missing data were addressed |
|  |  | (*d*) If applicable, explain how matching of cases and controls was addressed |
|  |  | (*e*) Describe any sensitivity analyses |
| Results | | |
| Participants | 13* | (a) Report numbers of individuals at each stage of study—eg numbers potentially eligible, examined for eligibility, confirmed eligible, included in the study, completing follow-up, and analysed |
|  |  | (b) Give reasons for non-participation at each stage |
|  |  | (c) Consider use of a flow diagram |
| Descriptive data | 14* | (a) Give characteristics of study participants (eg demographic, clinical, social) and information on exposures and potential confounders |
|  |  | (b) Indicate number of participants with missing data for each variable of interest |
| Outcome data | 15* | Report numbers in each exposure category, or summary measures of exposure |
| Main results | 16 | (*a*) Give unadjusted estimates and, if applicable, confounder-adjusted estimates and their precision (eg, 95% confidence interval). Make clear which confounders were adjusted for and why they were included |
|  |  | (*b*) Report category boundaries when continuous variables were categorized |
|  |  | (*c*) If relevant, consider translating estimates of relative risk into absolute risk for a meaningful time period |

**Supplementary Figures and Tables**

**481** Patient who underwent colostomy between 2013 and 2024

**260** patients excluded:

- 1. **214** Loop colostomy
  2. **46** Hartmann reversal

**221** Patient underwent definitive colostomy

**68** patients excluded:

- 1. **60** Insufficient follow up
  2. **8** Postoperative mortality

**56** received intervention (IPST funnel mesh)

n=56

**97** in control group (No IPST funnel mesh)

n=97

**56** included in analysis

**56** included in analysis

Propensity score matching

**Figure 1. Flow chart**

**Figure 2.** Kaplan–Meier estimates of parastomal hernia (PSH) - free survival prior to propensity score matching.

**Figure 3.** Kaplan–Meier estimates of parastomal hernia (PSH) - free survival after propensity score matching.
